# Microfluidic Chip for the Electrochemical Detection of MicroRNAs: Methylene Blue Increasing the Specificity of the Biosensor

**DOI:** 10.3389/fchem.2022.868909

**Published:** 2022-03-29

**Authors:** Claire Poujouly, Jérémy Le Gall, Martina Freisa, Djamila Kechkeche, David Bouville, Jihed Khemir, Pedro Gonzalez-Losada, Jean Gamby

**Affiliations:** Université Paris-Saclay, CNRS, Centre de Nanosciences et de Nanotechnologies, Palaiseau, France

**Keywords:** microfluidics, miRNA electrochemical detection, methylene blue (MB), microelectrodes, surface functionalization

## Abstract

MicroRNAs (miRNAs) are biomarkers involved in biological processes that are released by cells and found in biological fluids such as blood. The development of nucleic acid-based biosensors has significantly increased in the past 10 years because the detection of such nucleic acids can easily be applied in the field of early diagnosis. These biosensors need to be sensitive, specific, and fast in order to be effective. This work introduces a newly-built electrochemical biosensor that enables a fast detection in 30 min and, as a result of its integration in microfluidics, presents a limit of detection as low as 1 aM. The litterature concerning the specificity of electrochemical biosensors includes several studies that report one base-mismatch, with the base-mismatch located in the middle of the strand. We report an electrochemical nucleic acid biosensor integrated into a microfluidic chip, allowing for a one-base-mismatch specificity independently from the location of the mismatch in the strand. This specificity was improved using a solution of methylene blue, making it possible to discriminate a partial hybridization from a complete and complementary hybridization.

## Introduction

MicroRNAs are non-coding RNAs of 21 to 25 bases acting as regulators of protein translation. Since many diseases are caused by the misregulated activity of proteins, researchers actively studied microRNAs as biomarkers for early diagnosis of different types of cancer (Calin and Croce, 2006), as well as heart diseases (Ji et al., 2009; Adachi et al., 2010), and muscle damage (Siracusa et al., 2016; Siracusa et al., 2018). Standard methods for detecting microRNAs include northern blotting, microarrays, and real-time PCR (RT-PCR) (Cissell and Deo, 2009). However, these methods are time consuming and their limits of detection can be improved with electrochemical biosensors. Electrochemistry is an attractive technique in terms of sensitivity and ease of use (Drummond et al., 2003; Rosario and Mutharasan, 2014; Minaei et al., 2015). Electrochemical biosensors are based on converting a biological binding event to an electronic signal (Gooding, 2002; Grieshaber et al., 2008). In order to simplify the biosensor and reduce its number of detection steps, the direct detection of nucleic acids is generally favored. This direct detection is mostly based on the detection of the hybridization of two complementary strands of nucleic acids (Cissell and Deo, 2009). This hybridization induces a change in the electronic signal measured on the electrodes of the electrolytic cell. The change of signal can be generated by either direct charge transfer (Ariksoysal et al., 2005; Abbaspour and Noori, 2008), or by the phenomenon of long-range electron transfer (LET) (Kelley et al., 1999), playing an important role in the specificity of the biosensor (Boon et al., 2000). LET is based on the hypothesis that DNA-mediated electron transfer from an electrode to a redox mediator intercalated into the DNA duplex allows electrocatalysis of a redox tracer reduction in solution on DNA layers. For instance, Barton et al. (Boon et al., 2003a) described the electrocatalytic reduction of ferricyanide mediated by methylene blue (MB) using linear sweep voltammetry in a 3-electrode setup including a rotating gold electrode. The DNA duplexes (15-basis DNA with thiol linker) self-assembled into a dense monolayer in an upright position, blocking the electrochemical reduction. The authors reported that micromolar concentrations of the redox-active DNA intercalator are sufficient to enhance catalytic currents. In brief, the chemical reaction between MB and FeCN_6_^3−^ happens at the film/solution interface. The system needs a few seconds to reach a steady-state current density for FeCN_6_^3−^ reduction, which is a function of MB bulk concentration in the solution. For MB concentrations higher than 15 μM, they assumed that the reaction is supposed to be purely controlled by diffusion convection and its rate is proportional to the MB concentration in solution. To conclude, DNA films seem to be saturated with MB above 2 µM where binding of MB seems to be reversible with one intercalation site per 15-basis duplex. The specificity of the detection is one of the three most important key factors, together with the limit of detection and the rapidity, for nucleic acid biosensors to perform a reliable diagnosis (Horny et al., 2016; Nassi et al., 2016). Moreover, numerous electrode materials and electrochemical techniques can be used for sensitive measurements [cyclic voltammetry (Shoaie et al., 2018), differential pulse voltammetry (Butterworth et al., 2019), square wave voltammetry (Tsaloglou et al., 2018), and electrochemical impedance spectroscopy (Quan Li et al., 2017).

The integration of biosensors into microfluidics presents many advantages, such as reducing the number of samples needed as well as decreasing the time of experiments in parallel (Squires and Quake, 2005). In addition, microfluidic electrochemical nucleic acid biosensors enable a low detection threshold due to the diffusion layer's reduction under forced convection, bringing more nucleic acids to the sensor surface (Ferguson et al., 2009).

In order to resolve the above issues, we developed a microfluidic chip for the electrochemical detection of nucleic acids based on the LET using the system's catalytic nature to improve the sensor's specificity. We report an experimental study about enhancing the biosensor specificity by using methylene blue in a redox solution. We studied the specificity by evaluating the hybridization rate for target sequences with single-base mismatches in the middle or at the extremity of the nucleic acid strands. The intercalation of MB in double-stranded nucleic acids then enabled us to discriminate a partial hybridization from a complementary hybridization.

## Experimental

### Chemical Products and Nucleic Acids

Sodium chloride, potassium hexacyanoferrate (II) trihydrate, potassium hexacyanoferrate (III), and methylene blue (MB) were purchased from Sigma Aldrich. All aqueous solutions were prepared using deionized water with a resistivity of 18 MΩ. All DNA and RNA sequences were purchased from Eurogentec with reverse phase HPLC purification in a dried format. A 21-base thiol-labeled DNA probe (P) mimics the complementary sequence of the micro-ribonucleic acid 122 (miR 122). A DNA target (T) and an RNA target (T_RNA_) mimic the miR 122 sequence; these two targets are complementary to the DNA probe. A DNA target with one mismatch in the middle of the strand (T—1 M middle). A DNA target with one mismatch at the beginning of the strand (T—1 M beginning). A DNA target with one mismatch at the end of the strand (T—1 M end). A non-complementary DNA target (Nc T) mimics the miR 133-3p, involves muscle damage and serves as a negative test. Table 1 presents these sequences. The nucleic acids were first diluted in deionized water to obtain a 10^−4^ M concentration, and then diluted in a 0.5 M NaCl solution to get the wanted concentration.

**TABLE 1:** Nucleic acids sequences.

Name	Sequence
P	5′—Thiol modifier C6—CAA​ACA​CCA​TTG​TCA​CAC​TGC—3′
T	5′—GCA​GTG​TGA​CAA​TGG​TGT​TTG—3′
TRNA	5′—GCA​GUG​UGA​CAA​UGG​UGU​UUG—3′
T—1 M middle	5′—GCA​GTG​TGA​CCA​TGG​TGT​TTG—3′
T—1 M beginning	5′—GCA​GTG​TGA​CAA​TGG​TGT​TTT—3′
T—1 M end	5′—ACA​GTG​TGA​CAA​TGG​TGT​TTG—3′
Nc T	5′—TTT​GGT​CCC​CTT​CAA​CCA​GCT​G—3′

### Microfluidic Chip Fabrication

**FIGURE 1:**
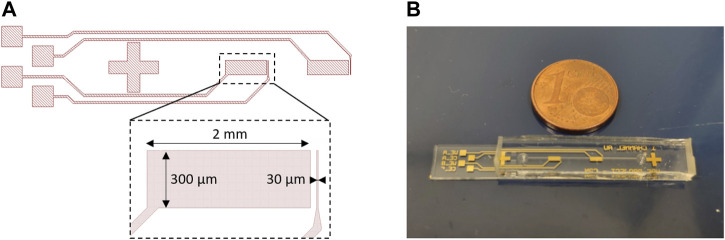
**(A)** Biosensor geometry, designed using KLayout software, with the detection area dimensions—working electrode (WE) (width 300 μm, length 30 µm), counter electrode (CE) (width 300 μm, length 2 mm). **(B)** Photography of the final microfluidic device composed of two pairs of gold electrodes (WE, CE) for repeatability of measurements.

The microfluidic device is formed by a glass substrate containing a pair of gold microelectrodes, and on top, a polydimethylsiloxane (PDMS) cover containing the microfluidic channel (300 µm wide, 60 µm high, 2 cm long). The pair of gold microelectrodes is composed of a working microelectrode (300 µm wide, 30 µm long) and a counter electrode (300 µm wide, 2 mm long) (Figure 1A). The microelectrodes were patterned onto a 4-inch glass wafer using a lift-off process. In order to realize the lift-off process, an initial photolithography step was realized using a negative photoresist (AZ nLOF 2020, MicroChemicals). Then we sputtered a 20 nm TiW layer on the whole wafer to improve the adhesion of the metal to the glass substrate. We completed the process by sputtering another 200 nm layer of Au. The process was completed by removing the photoresist in an acetone ultrasound bath for 15 mins, immediately followed by an isopropanol rinse. Once the electrodes were patterned, the wafer was diced into several chips. The microchannel structures were fabricated by molding PDMS on a SU-8 master mold already set on a silicon 4-inch wafer used as a substrate. For the mold fabrication the first layer of SU-8 2002 (MicroChemicals) of 2 µm was spin-coated on the wafer and baked to serve as an adhesion layer. Then a 60 µm layer of SU-8 2050 (MicroChemicals) was spin-coated, and microchannels were patterned by photolithography. Finally, we prepared a mixture of PDMS (RTV 615, Neyco), mixing 10 parts of silicone elastomer and one part of the curing agent. This mixture was then poured on the mold, placed under a vacuum for 2 h, and cured in an oven at 60°C for at least 4 h. After the PDMS was polymerized, it was peeled off from the mold and cut out. The inlet and outlet were punched using a biopsy needle (0.5 mm, Elveflow). At the end of the process, the diced electrode chips and the PDMS microchannels were bonded using oxygen plasma (Figure 1B). Once the electrode glass substrate and the PDMS microchannels are bonded, the device must be used within a week. If it is not used in this time period the effect of the oxygen plasma treatment on the PDMS microchannels disappears and the channels become hydrophobic. Considering the durability of the electrochemical sensor itself, once the first measurement is executed on the chip, all experiments must be carried out during the day. After that, the gold electrode surfaces start oxidizing.

### Experimental Protocol of Probe Immobilization and Target Hybridization

A syringe pump (neMESYS, Cetoni GmbH, Germany) and syringes of 1 to 5 ml are used to load the solutions into the microfluidic channel. The syringes are connected to the microfluidic channel via a Tygon tubing (1/16″ OD × 0.51 mm ID, Elveflow) and stainless steel couplers (23 G 0.025″ OD × 0.013″ ID, Elveflow) inserted into the inlet and outlet previously punched. The DNA probe immobilization was performed by loading the DNA probe solution, diluted to 10^−7^ M, into the microfluidic channel for 2 h. The thiol-modified DNA probe sequence spontaneously bound to the gold microelectrodes during the immobilization, forming the self-assembled monolayer (SAM) of DNA probes. Between the probe immobilization and the target recognition step, a 0.5 M NaCl solution was loaded into the microchannel for 30 min to test the stability of the SAM. The target hybridization was realized by loading into the microfluidic chip the nucleic acid target solution, containing the sequence of interest, for 30 min at a concentration varying from 10^−18^ M to 10^−6^ M. Electrochemical measurements were performed before and after this target hybridization step in order to determine the hybridization rate for the probe by comparing the measured currents.

### Electrochemical Measurements

An electrochemical workstation (Biologic SP-300, France) was used to record the electrochemical signals of the two-electrode system integrated into the microfluidic chip. At each step of electrochemical measurements (bare gold, ssDNA immobilization, dsDNA hybridization), the measured current can be attributed to the WE current response even if both the WE and the CE are functionalized with the probe sequence (Horny et al., 2016). EC-Lab software was used for the acquisition, processing, and display of all the electrochemical measurements. The cyclic voltammetry measurements were recorded from −0.2 to 0.2 V with a scan rate of 10 mV.s^−1^ in a 3 mM equimolar [Fe_(III)_ (CN)_6_]^3−^/[Fe_(II)_ (CN)_6_]^4−^ redox couple added to a 0.5 M NaCl solution. The chronoamperometry measurements were recorded at -0.2 V for 150 s in a 3 mM equimolar [Fe_(III)_ (CN)_6_]^3−^/[Fe_(II)_ (CN)_6_]^4−^ in a 0.5 M NaCl solution adding 5 µM of methylene blue solution. A micromolar concentration of MB is necessary to be electrochemically detected. Both electrochemical measurements were carried out at a flow rate of 0.5 μL/s. The flow rate was programmed using the syringe pump connected to the input of the microfluidic chip.

## Results and Discussion

The electrochemical system used consists of a two-electrode setup with a counter electrode (CE) surface area about 60-fold higher than the working electrode (WE) surface. Consequently, the CE current density variation is lower in comparison to the WE current density, allowing the CE to be considered as a pseudo-reference electrode. Therefore, the highest current density circulating in the electrolytic cell can be attributed to the WE current response.

All the experiments presented in this paper resulted from the functionalization of the WE with the complementary DNA probe for miR 122 capture, diluted at 10^−7^ M. The experimental section above describes the immobilization protocol leading to the DNA probe’s self-assembled monolayer (SAM). The specificity of the biosensor was tested with different sequences of nucleic acid targets in an equimolar solution of [Fe_(III)_ (CN)_6_]^3−^/[Fe_(II)_ (CN)_6_]^4−^ in NaCl with or without MB intercalation.

### Study of the Specificity in [Fe_(III)_ (CN)_6_]^3−^/[Fe_(II)_ (CN)_6_]^4−^

**FIGURE 2:**
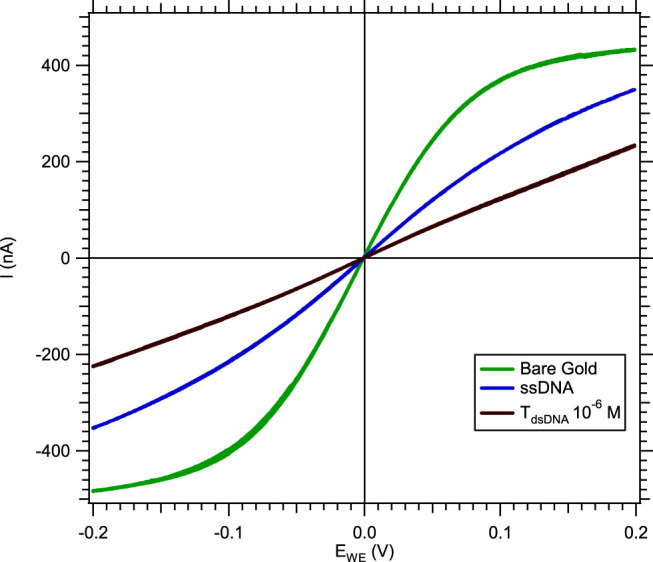
Cyclic voltammetry response of a microband WE in a solution of 3 mM [Fe_(III)_ (CN)_6_]^3−^/[Fe_(II)_ (CN)_6_]^4−^ and 0.5 M NaCl, with a 10 mV.s^−1^ scan rate and a 0.5 μL.s^−1^ flow rate, showing the current absolute value decreasing after hybridization.

In a solution of [Fe_(III)_ (CN)_6_]^3−^/[Fe_(II)_ (CN)_6_]^4−^ without MB, the hybridization of the nucleic acid target to the probe was deduced from the decreased current (in absolute value) compared to the measured current after single-stranded DNA-SAM formation (Figure 2).

The current decrease, measured by cyclic voltammetry, was due to the double-stranded nucleic acid limiting the electron transfer between the gold microelectrode and the redox tracer. To quantify the hybridization rate, we subtracted the current level of double-stranded nucleic acid (I_dsDNA_) from the current level of single-stranded DNA (I_ssDNA_). Since the gold microelectrodes' surface reproducibility depends on external conditions during the microfabrication process in the clean room, the current level of different microelectrodes might present certain differences. In order to compare all electrochemical measurements, a normalization procedure was systematically applied to the data by plotting the difference between I_ssDNA_–I_dsDNA_ divided by the ssDNA current level (Eq. 1).

$$I_{\mathrm{hyb}}=\frac{\left|I_{\mathrm{ssDNA}}-I_{\mathrm{dsDNA}}\right|}{\left|I_{\mathrm{ssDNA}}\right|}\;$$ (1)

I_ssDNA_ is the current level after immobilization of the DNA probe as SAM on the WE, and I_dsDNA_ is the current level after loading the nucleic acid target, both selected at −0.2 V on the voltammograms.

**FIGURE 3:**
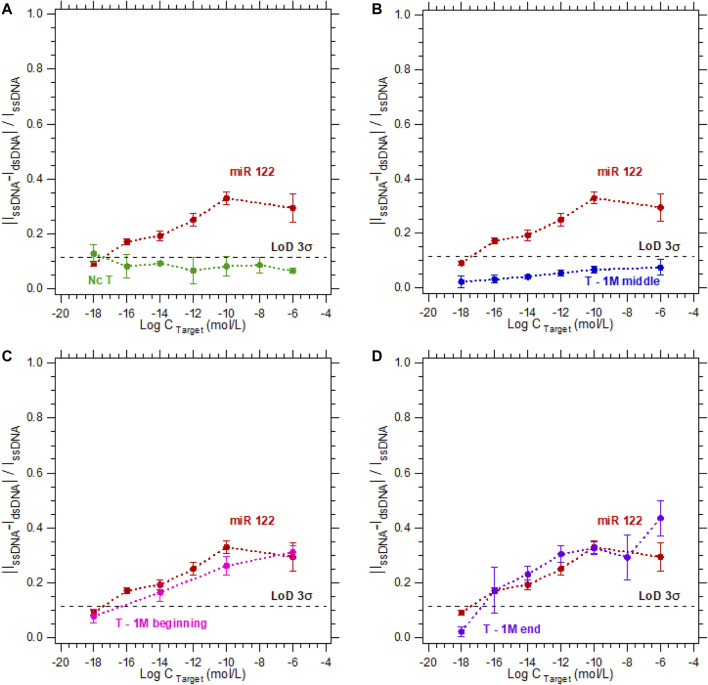
Calibration curves in 3 mM [Fe_(III)_ (CN)_6_]^3−^/[Fe_(II)_ (CN)_6_]^4−^ and 0.5 M NaCl for the detection of DNA target at concentrations varying from 10^−18^ M to 10^−6^ M. For a DNA target complementary to the DNA probe sequence (red dots—called miR 122) compared to the calibration curves of **(A)** a non-complementary target (green dots), **(B)** a sequence with a one-base mismatch located in the middle (blue dots), **(C)** at the beginning (pink dots), **(D)** or at the end (purple dots) of the strand.

To study the specificity of the electrochemical microfluidic chip, we tested different target sequences. The five sequences we focused on were a complementary sequence, a non-complementary sequence, and a sequence with a 1-base mismatch at different locations in the strand (at the beginning, in the middle, or at the end). The target concentration varied from 10^−18^ to 10^−6^ M. The hybridization detection was analyzed according to Eq. 1, and plotted as a calibration curve. The limit of detection (LoD) 3σ is equal to 0.115 was calculated using the definitions established by Armbruster and Pry (2008). In Figure 3A, the probe and its complementary target (duplex named miR-122) serve as a reference for all hybridization tests since they can be considered as the perfect hybridization match. The hybridization detection experiment between the probe and the non-complementary target (named Nc T) served as a control test since no hybridization is expected (Figure 3A). As shown in Figure 3B, the sensor is highly specific regarding the target sequences with a 1-base mismatch when the mismatch is in the middle of the strand (experiment named T—1 M middle). The calibration curve is below the LoD 3σ, meaning there was no detection from the sensor for this sequence. In contrast when the single-base mismatch is located at the beginning of the strand (experiment named T—1 M beginning, Figure 3C) or at the end of the strand (experiment named T—1 M end, Figure 3D), the discrimination between these experiments and the complementary one is impossible. This inability to discriminate can be explained by a partial hybridization of the two strands, even if the sequences are not perfectly complementary. In these cases, the double-stranded nucleic acid limits the electron transfer between the electrode and the electrolyte as efficiently as a complementary hybridization. Consequently, in a ferri/ferrocyanide solution without a DNA intercalator, the sensor does not enable discrimination of a partial hybridization (T—1 M beginning or T—1 M end) from a complementary hybridization with no mismatch.

### Study of the Specificity in [Fe_(III)_ (CN)_6_]^3−^/[Fe_(II)_ (CN)_6_]^4−^and Methylene Blue

**FIGURE 4:**
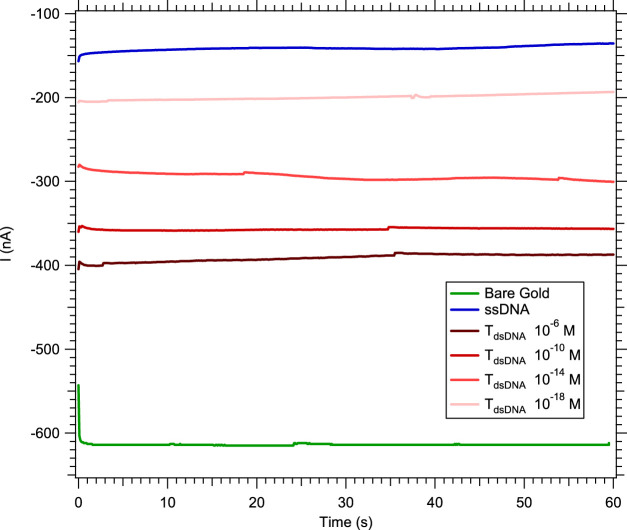
Chronoamperometry at −0.2 V in 3 mM [Fe_(III)_ (CN)_6_]^3−^/[Fe_(II)_ (CN)_6_]^4−^ and 0.5 M NaCl with 5 µM MB and a 0.5 μL.s^−1^ flow rate, showing the absolute value of the current increasing with the DNA target concentration.

In order to improve the specificity of the sensor by distinguishing the hybridization detection of a single-base mismatch (independently of its location in the strand) from a complementary hybridization, we repeated the previous experiments in a solution of [Fe_(III)_ (CN)_6_]^3−^/[Fe_(II)_ (CN)_6_]^4−^ in NaCl with MB. Methylene blue is well-known for its intercalation in double-stranded nucleic acids (Tuite and Norden, 1994; Kelley et al., 1997; Boon et al., 2003a; Boon et al., 2003b; Nafisi et al., 2007; Furst et al., 2015; Kékedy-Nagy and Ferapontova, 2018; Furst et al., 2019). In this electrocatalytic process, electrons pass from the electrode surface to the intercalated MB^+^. Leucomethylene blue (LB^+^), the reduced form of MB^+^, reduces the ferricyanide solution, allowing the catalytic cycle to continue (Boon et al., 2000). Due to the catalytic nature of the system, the overall electrocatalytic response increases with the integration times. Therefore, the hybridization of the nucleic acid target to the probe was measured by chronoamperometry. In the case of double-stranded nucleic acids, more MB^+^ molecules are electrochemically reduced, which increases the concentration of active catalyst, thus increasing the absolute value of the current level measured after hybridization (Figure 4).

For these reasons, the normalization procedure explained above for Eq. 1 is modified to quantify the hybridization, by subtracting I_ssDNA_ from I_dsDNA_ and normalizing by I_dsDNA_ (Eq. 2) as follows:

$$I_{hyb}=\frac{\left|I_{dsDNA}-I_{ssDNA}\right|}{\left|I_{dsDNA}\right|}$$ (2)

**FIGURE 5:**
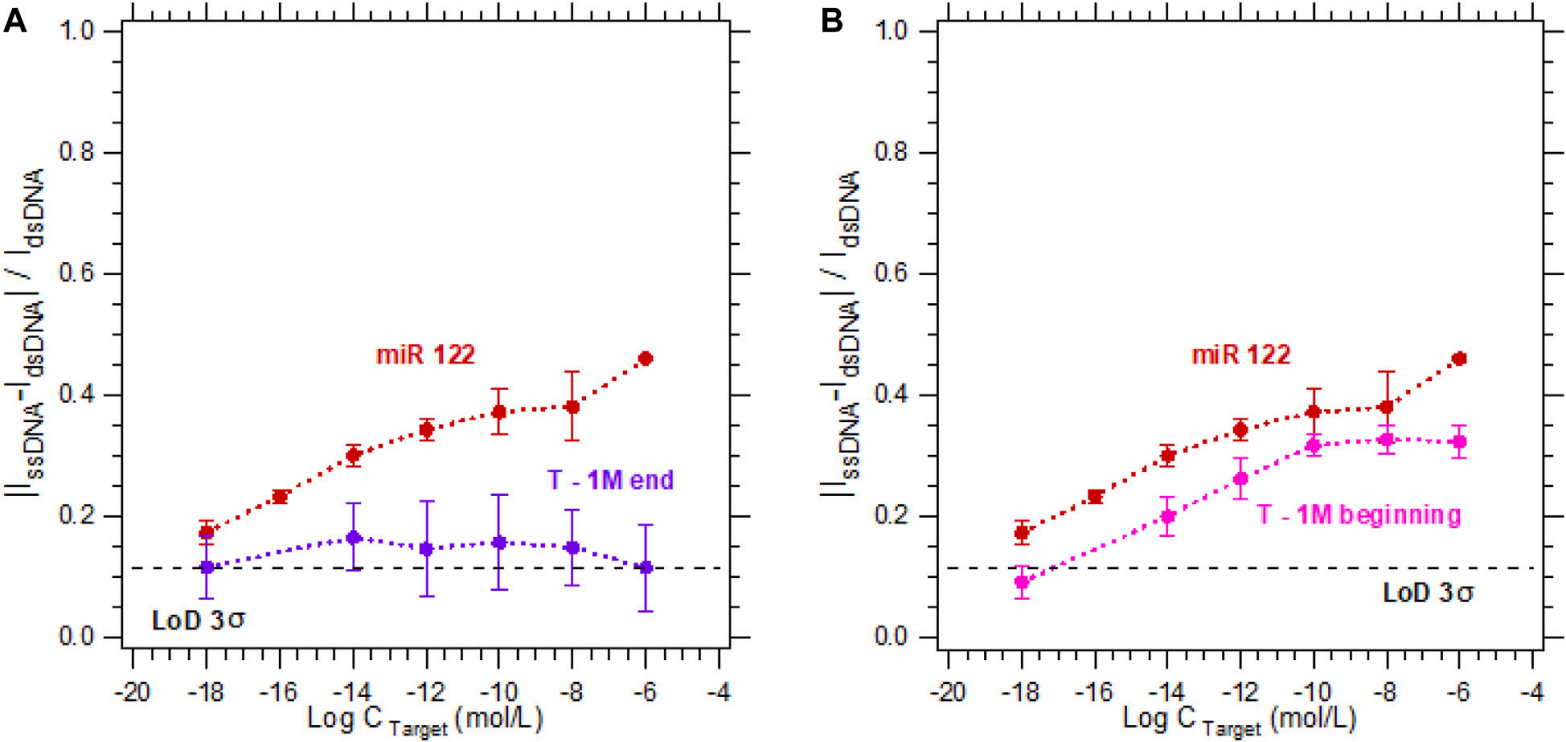
Calibration curves in 3 mM [Fe_(III)_ (CN)_6_]^3−^/[Fe_(II)_ (CN)_6_]^4−^ and 0.5 M NaCl with 5 µM MB for the detection of DNA target at concentrations varying from 10^−18^ M to 10^−6^ M. For a DNA target complementary to the DNA probe sequence (red dots—called miR 122) compared to the calibration curves of a sequence with a one-base mismatch located **(A)** at the end (purple dots), or **(B)** at the beginning (pink dots) of the strand.

In Figure 5, the probe and its complementary target (duplex named miR 122) serve as a reference for all hybridization tests. As presented on Figure 5, when using 5 µM MB in a 3 mM [Fe_(III)_ (CN)_6_]^3−^/[Fe_(II)_ (CN)_6_]^4−^ solution for the detection of a single-mismatch located at the end of the strand, the calibration curve obtained is independent from the increasing targets concentration. Indeed, since the mismatch is far from the electrode surface, and even if the strands are partially hybridized, the electrons cannot flow from the electrode to the electrolyte. In the opposite case, for a mismatch at the beginning of the strand, electrons can pass from the electrode to the electrolyte more easily than for a mismatch at the end of the strand, resulting in an increase in the hybridization current. However, this current is still lower than that for the complementary strand, and can be distinguished from the complementary target by a concentration of the complementary target higher than 10^−12^ M.

**FIGURE 6:**
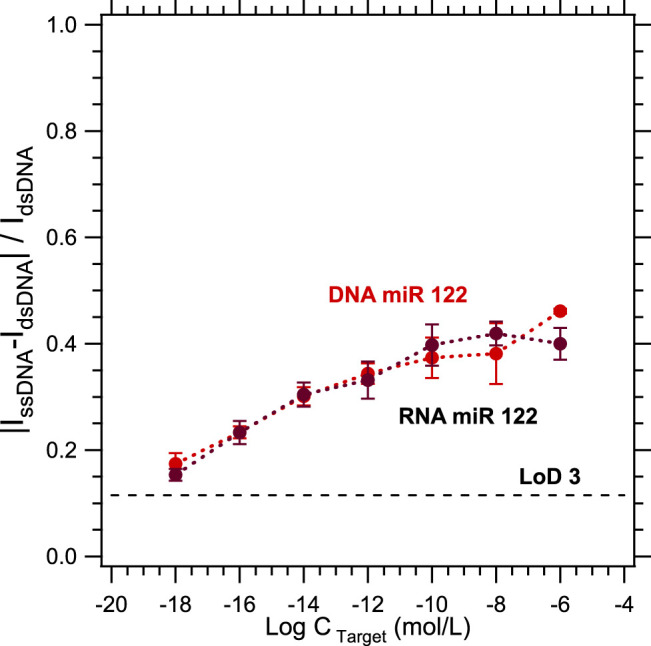
Calibration curves for the hybridization detection comparison between DNA/DNA duplex (red dots) and DNA/RNA duplex (black dots).

The next step will be to test the specificity of the sensor in a biological blood sample. In a biological sample, the sequence of interest is a micro-ribonucleic acid, while until now, the sequences studied were deoxyribonucleic acids. In this context, an intermediate step towards real blood samples was realized: the detection of DNA/DNA duplexes versus DNA/RNA duplexes was compared. Figure 6 compares the calibration curves for the detection of a DNA target sequence and an RNA target sequence, both complementary to the same DNA probe sequence. The current levels measured being roughly the same, it underlines that there is no difference between the detection of a DNA target or an RNA target. As a result, the sensor's specificity results could be suitable for a blood sample analysis, for which a pre-treatment with commercially available miRNA extraction kits should be necessary.

## Conclusion

In conclusion, we developed a nucleic acid biosensor composed of an electrochemical cell integrated into a microfluidic chip, enabling the detection of a microRNA sequence in 30 min. Its specificity was improved by adding methylene blue (MB), a nucleic acid intercalator electrochemically active. Without MB, the electrochemical detection of a partial hybridization is indiscernible from the detection of a complete and complementary hybridization. By using MB at a micromolar concentration, the possibility of discriminating between these two types of hybridization was demonstrated, and the specificity of the biosensor improved, independent of the location of the mismatch in the sequence. The value of 1 a.m. is the LoD for two complementary strands. However, when the mismatch is located at the beginning of the strand, and for concentrations below 1 p.m., the calibration curve measured does not enable discrimination between this kind of mismatch and the perfect match, leading to the biosensor’s sensitivity being limited to 1 p.m. Moreover, this protocol is promising for the electrochemical detection of nucleic acids in biological samples.

## Data Availability

The original contributions presented in the study are included in the article/Supplementary Material; further inquiries can be directed to the corresponding author.
